# Immune regulation in mitochondrial transfer: knowledge structure and emerging trends from a bibliometric perspective

**DOI:** 10.3389/fimmu.2026.1850600

**Published:** 2026-06-26

**Authors:** Qiyu He, Zhimin Tan, Juan Xu, Tianhong Wang, Qian Li, Xiaoqiang Li

**Affiliations:** 1 Department of Urology, West China Hospital of Sichuan University, Chengdu, China; 2 Department of Anesthesiology, Laboratory of Mitochondria and Metabolism, West China Hospital, Sichuan University, Chengdu, China; 3 Operating Room, West China Hospital, Sichuan University/West China School of Nursing, Sichuan University, Chengdu, China; 4 National Health Commission of the People’s Republic of China (NHC) Key Laboratory of Clinical Epidemiology and Evidence-based Medicine, West China Hospital, Sichuan University, Chengdu, China; 5 Sichuan Center of Technology Innovation for Real World Data, Chengdu, China; 6 Department of Anesthesiology and Operation Center, West China Tianfu Hospital, Sichuan University, Chengdu, China

**Keywords:** bibliometric analysis, immune regulation, immunometabolism, mitochondrial transfer, mitochondrial transplantation

## Abstract

**Background:**

Mitochondrial transfer has emerged as an important form of intercellular communication with growing relevance to immune regulation, inflammation, tissue repair, and tumor immunity. However, the knowledge structure, developmental trajectory, and emerging hotspots of this field remain unclear.

**Methods:**

We conducted a bibliometric analysis of studies on mitochondrial transfer and immune regulation published between 2016 and 2025. Publications were retrieved from PubMed, Embase, the Cochrane Library, Scopus, and Web of Science, and analyzed using bibliometrix in R and CiteSpace. Annual publication trends, contributions of countries, institutions, authors, and journals, as well as keyword co-occurrence, clustering, burst detection, and co-citation patterns were evaluated.

**Results:**

A total of 967 publications were included. Annual publication output increased steadily, with faster growth after 2020. China and the United States were the leading contributors and occupied central positions in international collaboration networks. Keyword and co-citation analyses showed that early studies mainly focused on mitochondrial DNA-associated inflammatory sensing, innate immunity, and inflammatory injury, whereas recent studies increasingly emphasized intercellular mitochondrial transfer, mitochondrial transplantation, T-cell function, tumor-associated macrophages, cancer immunotherapy, metabolic rewiring, and autophagy-associated mitochondrial quality control. Mitochondrial transplantation and tunneling nanotube were among the most prominent burst terms. Co-citation analysis identified major knowledge domains related to mitochondrial danger signaling, intercellular transfer mechanisms, mesenchymal stem cell-mediated immune regulation, tumor immunity, and translational applications.

**Conclusion:**

Bibliometric mapping shows a clear shift from mitochondrial danger signaling toward intercellular transfer and immune-cell metabolic remodeling. Current evidence suggests that immune outcomes are shaped by mitochondrial source, transfer route, recipient-cell state, and disease context. More source-defined and context-specific studies are needed to clarify the therapeutic potential of mitochondrial transfer.

## 1 Introduction

Mitochondria have traditionally been regarded as intracellular organelles that support energy metabolism, biosynthesis, and signal integration within the host cell (1). Recent evidence have shown that under specific physiological or pathological conditions, mitochondria can be transferred between cells while retaining a degree of structural integrity and metabolic activity in recipient cells (2–4). This phenomenon has been observed in tissue repair, the tumor microenvironment, inflammatory responses, and models of systemic disease (5–12). Tunneling nanotubes, extracellular vesicles, cell fusion, and mitochondrial release and uptake have all been implicated as potential routes of intercellular mitochondrial transfer (3, 13–18). These findings indicate that mitochondria can participate in microenvironmental homeostasis and disease progression through intercellular transfer.

Immune cell differentiation, activation, and effector function are closely linked to mitochondrial state, and different immune cell subsets show substantial differences in mitochondrial dynamics, bioenergetic demand, and metabolic programs (19–23). Intercellular mitochondrial transfer can improve oxidative phosphorylation and survival in recipient cells, and can further alter immune cell metabolism and functional state, thereby contributing to inflammation, immune suppression, and tissue repair (13–16, 24). Mitochondria also represent an important source of immune signals. Mitochondrial DNA, reactive oxygen species, and N-formyl peptides can be sensed by the innate immune system as damage-associated molecular patterns and activate multiple inflammatory signaling pathways (25–27). As this field has developed, the role of mitochondria in immune regulation has come to include changes in recipient-cell metabolism and the immune microenvironment, particularly in cancer, inflammatory injury, and stem cell-mediated repair.

Despite the rapid growth of the literature, the overall knowledge structure of this field remains incompletely defined. Current studies often discuss mitochondrial transfer, mitochondrial transplantation, mtDNA-associated inflammatory sensing, immunometabolic regulation, and functional observations in specific disease models together, although these topics do not fully share the same scope or central questions. As the field has expanded, it has become increasingly important to clarify whether the main emphasis remains on inflammatory sensing or has moved toward intercellular transfer, metabolic remodeling, and microenvironmental change. Existing reviews have provided important insights into individual mechanisms and disease contexts, but a quantitative overview of the field’s knowledge base, thematic changes, and emerging directions is still lacking.

Bibliometric analysis provides a quantitative approach to characterizing the knowledge structure and development of a research field through publication trends, keyword co-occurrence, co-citation patterns, and burst detection (28, 29).

On this basis, we performed a bibliometric analysis of research on mitochondrial transfer and immune regulation to define the field’s knowledge structure, trace thematic changes, and to identify major topics and emerging directions that may inform future mechanistic and translational studies.

## 2 Methods

### 2.1 Data source and search strategy

A systematic search was conducted in PubMed, Embase, the Cochrane Library, Scopus, and Web of Science for studies related to mitochondrial transfer and immune regulation, covering the period from January 1, 2016 to December 31, 2025. All searches and data exports were completed on the same day. The search strategy combined controlled vocabulary and free-text terms, and the full search strings are provided in **Supplementary Table 1**.

**Figure 1:**
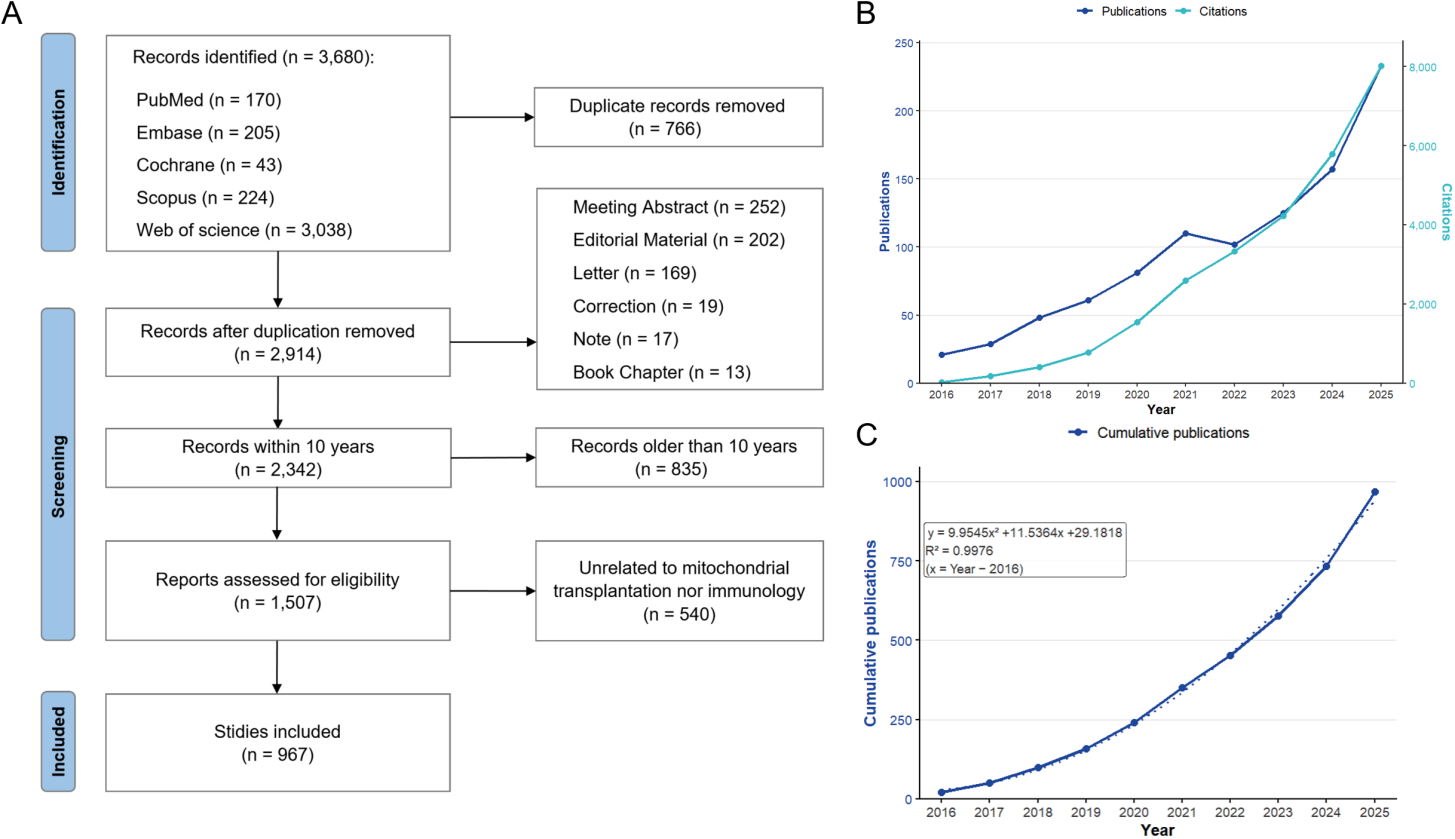
Literature selection process and publication and citation trends in the field of mitochondrial transfer and immunology (2016–2025). **(A)** Flowchart of literature inclusion and exclusion. **(B)** Annual trends of publications and citations. **(C)** Descriptive polynomial trend line for cumulative publication output from 2016 to 2025.

Publications were included if they focused on mitochondrial transfer and immune regulation and were indexed as original articles or reviews. Duplicates, conference abstracts, editorials, letters, and articles unrelated to the topic were excluded (**Figure 1A**). Study selection followed the PRISMA statement (30). Titles, abstracts, and full texts were screened independently by two reviewers. Disagreements were resolved through discussion, and unresolved cases were adjudicated by a third reviewer. A total of 967 publications were included in the bibliometric analysis.

### 2.2 Data extraction and preprocessing

Records were exported in “Full Record and Cited References” format as plain text files. Information on authors, titles, journals, publication year, countries or regions, affiliations, keywords, references, and citations was retained. Data from all databases were imported into EndNote 2025 (Clarivate Analytics, USA) for merging, deduplication, and standardization, yielding the final dataset for analysis. To reduce inconsistencies across databases, bibliographic fields and cited references were standardized before network construction and visual analysis.

### 2.3 Bibliometric analysis

Bibliometric analyses were performed using R with the bibliometrix package (v4.3.1) and CiteSpace (v6.2.R4) (28, 29, 31, 32). Bibliometrix was used for descriptive analyses, annual publication trends, and contributions across countries, institutions, authors, and journals, as well as for collaboration network visualization. CiteSpace was used for keyword co-occurrence, clustering, burst detection, and co-citation analysis. The resulting maps were interpreted together with representative highly cited, co-cited, and cluster-defining studies, allowing the main bibliometric signals to be linked with transfer routes, recipient-cell responses, and disease-context evidence.

To further describe publication growth, cumulative publication output was displayed with a polynomial trend line. This curve was used only for visualization of the observed growth pattern during the included study period and was not used for prediction. The analysis covered 2016 to 2025 with 1-year time slices. Nodes included countries or regions, institutions, authors, keywords, and cited references. Cluster labels were generated using the log-likelihood ratio method, and cluster quality was assessed by modularity Q and mean silhouette values. Burst detection followed the Kleinberg algorithm.

### 2.4 Network visualization and knowledge mapping

Collaboration was analyzed at the level of countries, institutions, and authors, with nodes representing entities and links indicating collaborative relationships. Keyword co-occurrence was used to outline major themes, and co-citation analysis to reflect the underlying knowledge base. In CiteSpace, nodes were selected using the g-index and networks were pruned using the Pathfinder algorithm. Node size reflects frequency or citation counts, and links represent co-occurrence or co-citation relationships, with color indicating temporal distribution. Timeline views based on clustering illustrate the emergence and progression of major themes.

## 3 Results

### 3.1 Annual publication and citation trends

From 2016 to 2025, publication output in mitochondrial transfer and immune regulation showed a steady increase. The 967 included studies accumulated 18,642 citations, with an average of 19.28 citations per article and an H-index of 61. Annual publication growth was modest between 2016 and 2019, followed by a sustained rise after 2020, with a more pronounced increase from 2022 onward (**Figure 1B**). To further describe the growth of the field, cumulative publication output was displayed using a descriptive polynomial trend line (y = 9.9545x² + 11.5364x + 29.1818, R² = 0.9976; x = Year − 2016; **Figure 1C**).

### 3.2 Contributions of countries and institutions

**Figure 2:**
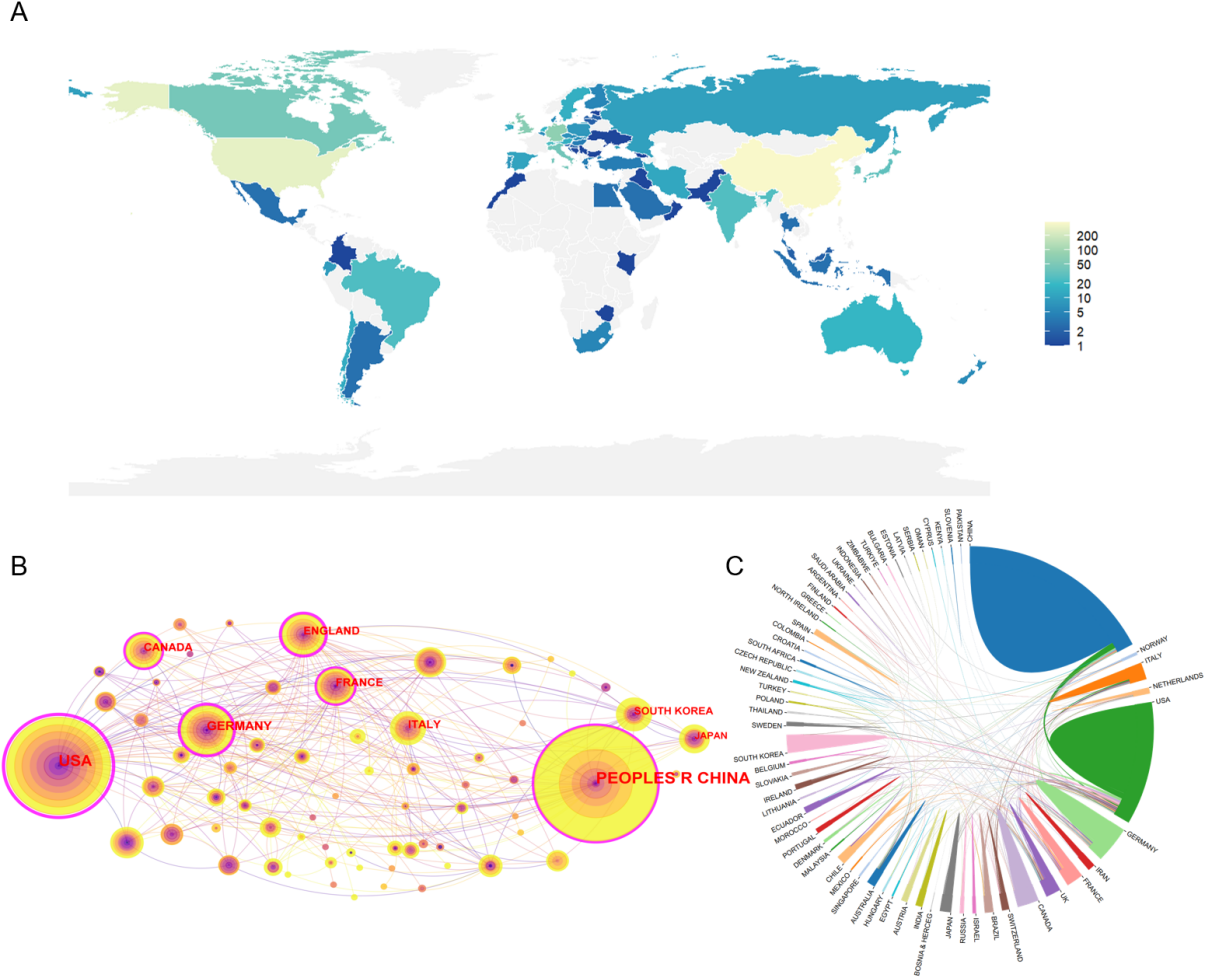
National collaboration and distribution in the field of mitochondrial transfer and immunology (2016–2025). **(A)** Co-occurrence network of major countries. **(B)** Sankey diagram showing the publication quantity distribution and collaboration relationships among countries. **(C)** Global distribution of publication quantity by country.

Research activity is largely centered in North America, East Asia, and Europe (**Figure 2A**). China and the United States dominate both in output and in the extent of international collaboration, occupying central positions in the global network (**Figure 2B**). A broader group of countries, including Germany, the United Kingdom, France, Italy, Japan, South Korea, and Canada, are also well integrated into this network and maintain close connections with the leading contributors (**Figure 2C**).

**Figure 3:**
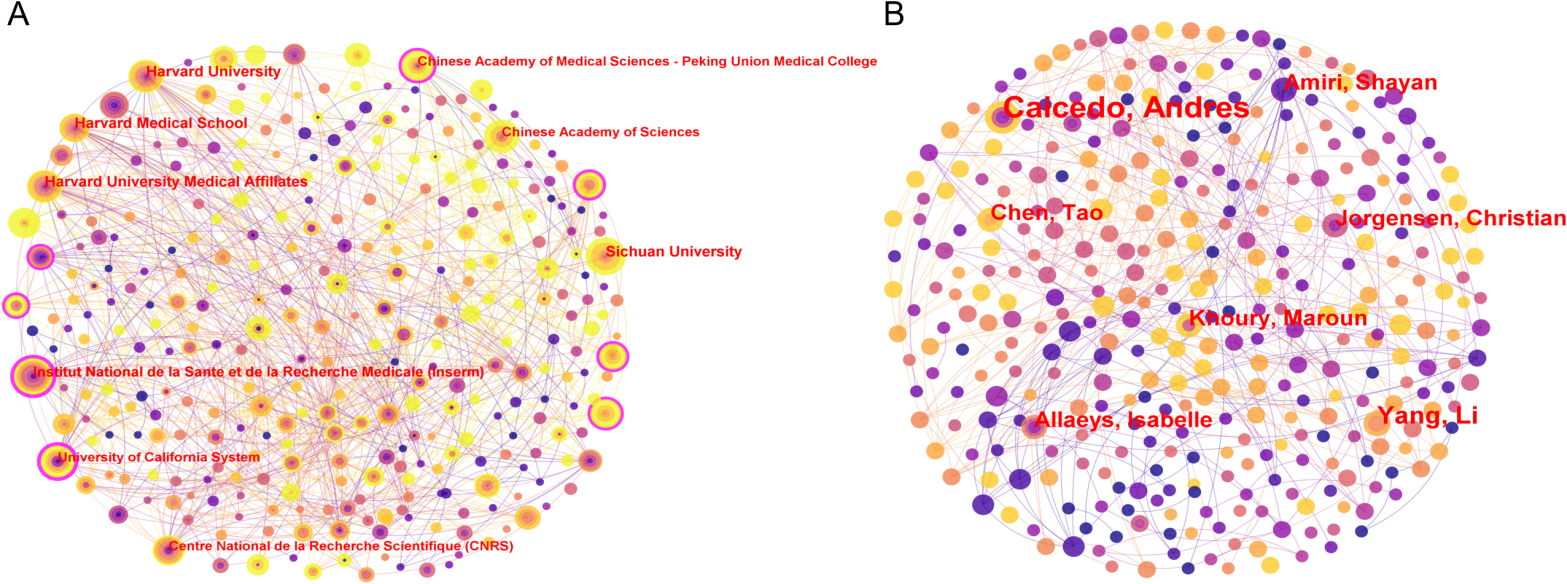
Author and institution collaboration networks in the field of mitochondrial transfer and immunology (2016–2025). **(A)** Institution collaboration network. **(B)** Author collaboration network.

At the institutional level, contributions are mainly driven by major universities and medical research centers. Sichuan University leads in publication count, followed by Harvard Medical School and the University of Pittsburgh, with several other institutions contributing at a comparable scale (**Supplementary Table 2**). Collaboration patterns indicate a network organized around these high-output institutions, with interactions concentrated among a relatively small set of core nodes (**Figure 3A**).

### 3.3 Author collaboration, journals, and funding sources

The author collaboration network showed that the field has formed several relatively stable research groups. Among the most productive authors were Caicedo A, Khoury M, Luz-Crawford P, and Boilard E (**Figure 3B**; **Supplementary Table 3**). The included studies were published mainly in journals in immunology, cell biology, materials science, and translational medicine. *Frontiers in Immunology*, *International Journal of Molecular Sciences*, *Advanced Science*, *Biomaterials*, *Cells*, and *Nature Communications* were among the most active publication venues (**Supplementary Table 4**). Funding was derived mainly from national research agencies and public health funding systems. The National Natural Science Foundation of China, the US Department of Health and Human Services, and the National Institutes of Health were the leading funding sources in this field (**Supplementary Table 5**).

### 3.4 Keyword co-occurrence and research topics

**Figure 4:**
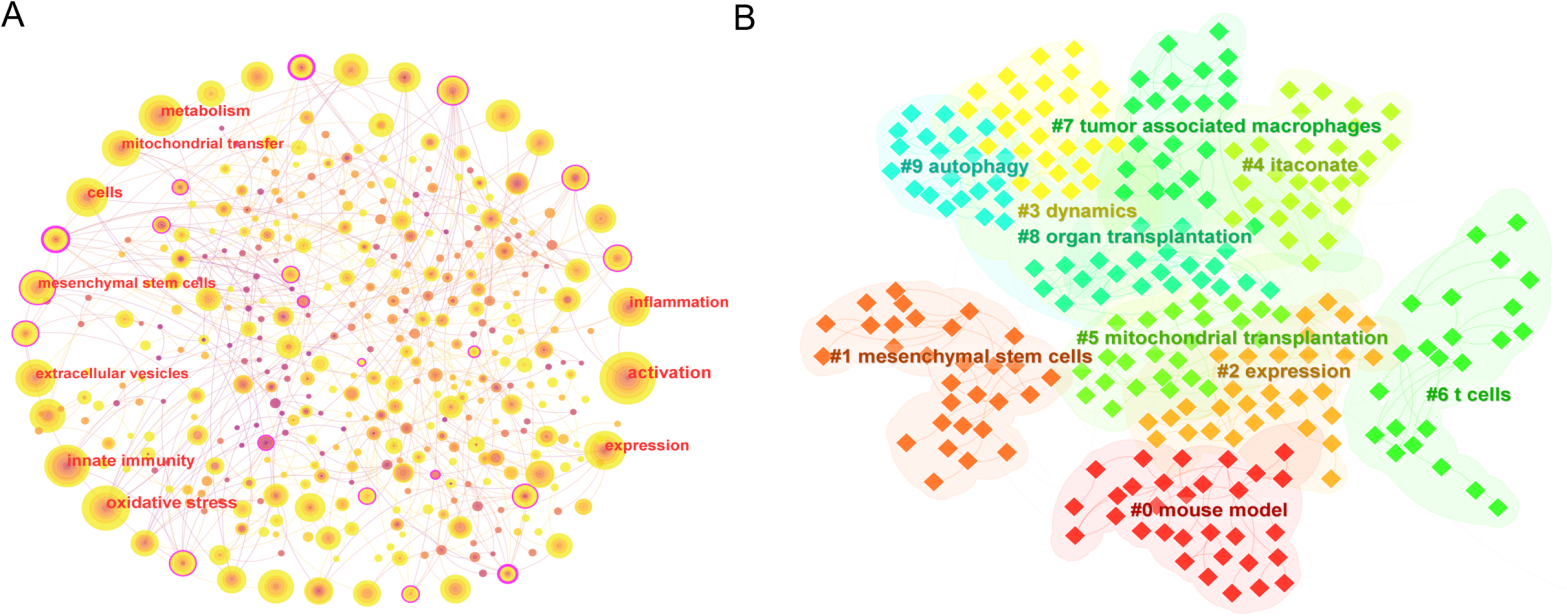
Keyword co-occurrence and topic clustering in the field of mitochondrial transfer and immunology (2016–2025). **(A)** Keyword co-occurrence network. **(B)** Topic clustering of keyword.

The keyword co-occurrence network identified “mitochondrial transfer,” “innate immunity,” “inflammation,” “activation,” “oxidative stress,” “extracellular vesicles,” and “mesenchymal stem cells” as high-frequency terms. Taken together, high-frequency keywords concentrated on transfer routes, delivery carriers, and the immune or inflammatory consequences of mitochondrial exchange, with substantial overlap with studies on extracellular vesicles and mesenchymal stem cells (**Figure 4A**).

Keyword clustering revealed several related research areas within the field (**Figure 4B**). Experimental and inflammatory injury studies were mainly represented by the #0 mouse model cluster, while the #1 mesenchymal stem cells cluster captured MSC-associated mitochondrial transfer and immune modulation. A more intervention-oriented branch was reflected by the #5 mitochondrial transplantation cluster, which included studies of exogenous mitochondrial delivery and related applications. Immune-cell and tumor-microenvironment topics were concentrated in the #6 T cells and #7 tumor-associated macrophages clusters. The #9 autophagy cluster appeared in a mitochondrial quality-control context, with links to mitochondrial turnover, organelle stress, and intracellular remodeling after mitochondrial injury or transfer. It was therefore interpreted as an adjacent quality-control topic, not as a transfer-route cluster or direct evidence of mitochondrial transfer itself.

### 3.5 Temporal evolution and burst analysis

**Figure 5:**
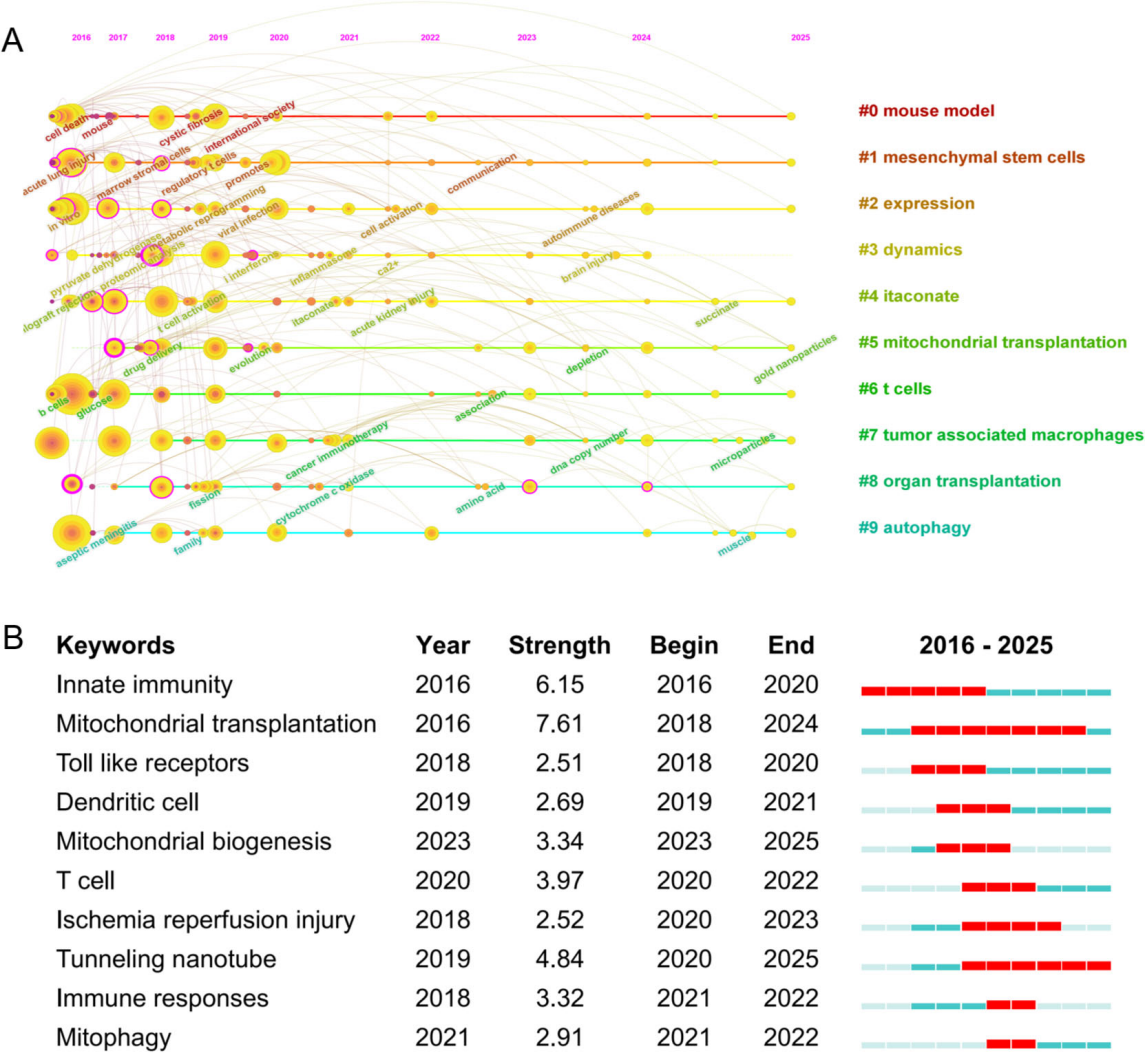
Keyword evolution and burst analysis in the field of mitochondrial transfer and immunology (2016–2025). **(A)** Time-based keyword evolution analysis. **(B)** Keyword burst analysis.

Timeline analysis showed a gradual shift in topic emphasis from inflammatory injury and MSC-related models to transfer routes, immune-cell function, tumor immunity, and mitochondrial quality control (**Figure 5A**). Early activity was concentrated in the #0 mouse model and #1 mesenchymal stem cells clusters, with keywords such as cell death, acute lung injury, bone marrow stromal cell, regulatory T cells, and T-cell activation. These terms reflected early work on inflammatory injury models and MSC-mediated immune modulation. Later activity extended toward intercellular communication and intervention-oriented topics, especially in the #5 mitochondrial transplantation and #8 organ transplantation clusters, with terms such as communication, cell activation, inflammation, mitochondrial transplantation, extracellular mitochondria, and organ transplantation. Recent themes were more closely related to immune-cell function, tumor immunity, and mitochondrial quality control. The #6 T cells and #7 tumor-associated macrophages clusters included terms such as T cells, cancer immunotherapy, and tumor-associated macrophages, whereas the #9 autophagy cluster appeared together with mitophagy, mitochondrial biogenesis, depletion, and DNA copy number, indicating increased attention to mitochondrial turnover and intracellular fate after mitochondrial stress or transfer. These autophagy-related terms were interpreted as quality-control and intracellular-fate signals, not as direct transfer-route terms.

Burst analysis further captured shifts in research emphasis across different periods (**Figure 5B**). “Mitochondrial transplantation” showed the strongest burst, with a strength of 7.61 from 2018 to 2024, and was also among the longest-lasting burst terms. “Innate immunity” ranked second, with a burst strength of 6.15 from 2016 to 2020. “Tunneling nanotube” showed a burst strength of 4.84 from 2020 to 2025 and remained active in recent years. “T cell,” “Mitochondrial biogenesis,” and “Immune responses” showed burst strengths of 3.97, 3.34, and 3.32, respectively. In addition, “Mitophagy,” “Ischemia reperfusion injury,” “Dendritic cell,” and “Toll like receptors” also showed notable bursts at different time points. Burst patterns suggest an early emphasis on innate immune sensing and inflammatory injury, followed by growing attention to intercellular mitochondrial transfer, immune-cell functional remodeling, and mitochondrial quality control in more recent years.

### 3.6 Co-citation structure and knowledge base

**Figure 6:**
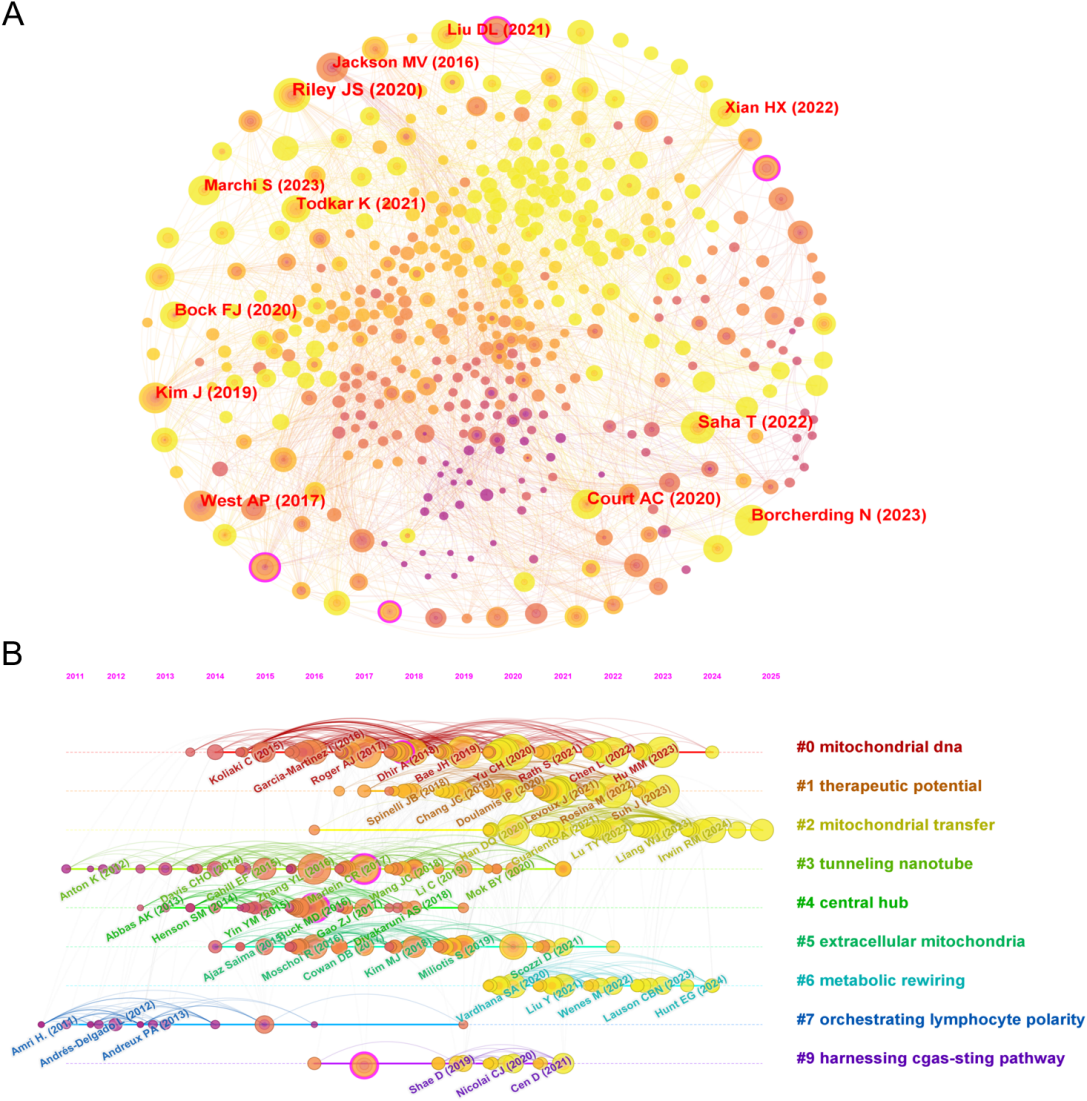
Co-citation network and evolution analysis in the field of mitochondrial transfer and immunology. **(A)** Co-citation network analysis. **(B)** Co-citation evolution analysis.

Co-citation network analysis identified several relatively distinct knowledge domains in this field, including mtDNA-related immune inflammation, intercellular mitochondrial transfer, mesenchymal stem cell mediated immune regulation, and the potential applications of mitochondrial transfer (**Figure 6A**). Among the highly cited representative studies, Riley JS (2020) (33) focused on the relationship between mtDNA and immune inflammation, Saha T (2022) (7) examined tunneling nanotube mediated mitochondrial transfer between cancer cells and immune cells, and Court AC (2020) (34) reported that mitochondrial transfer from MSCs to T cells promoted Treg differentiation and reduced inflammation (**Table 1**).

**Table 1:** Top ten highly co-cited and cited studies on mitochondrial transfer and immunology.

Top ten highly co-cited articles								
Rank	Author	Year	Journal	IF	JCR	Country	Co-cited	Title
1	Riley JS	2020	EMBO reports	6.2	Q1	U.K.	38	Mitochondrial DNA in inflammation and immunity (33)
2	Saha T	2022	Nature Nanotechnology	35.1	Q1	U.S.	31	Intercellular nanotubes mediate mitochondrial trafficking between cancer and immune cells (7)
3	Court AC	2020	EMBO reports	6.2	Q1	Chile	30	Mitochondrial transfer from MSCs to T cells induces Treg differentiation and restricts inflammatory response (34)
4	Borcherding N	2023	Nature	48.5	Q1	U.S.	27	The power and potential of mitochondria transfer (2)
5	Bock FJ	2020	Nature Reviews Molecular Cell Biology	90.2	Q1	U.K.	26	Mitochondria as multifaceted regulators of cell death (35)
6	Todkar K	2021	Nature Communications	15.7	Q1	Canada	26	Selective packaging of mitochondrial proteins into extracellular vesicles prevents the release of mitochondrial DAMPs (36)
7	Kim J	2019	Science	45.8	Q1	U.S.	25	VDAC oligomers form mitochondrial pores to release mtDNA fragments and promote lupus-like disease (37)
8	West AP	2017	Nature Reviews Immunology	60.9	Q1	U.S.	25	Mitochondrial DNA in innate immune responses and inflammatory pathology (25)
9	Marchi S	2023	Nature Reviews Immunology	60.9	Q1	U.S.	24	Mitochondrial control of inflammation (38)
10	Liu D	2021	Signal Transduction and Targeted Therapy	52.7	Q1	China	24	Intercellular mitochondrial transfer as a means of tissue revitalization (5)
Top ten highly cited articles								
Rank	Author	Year	Journal	IF	JCR	Country	Cited	Title
1	Riley JS	2020	EMBO reports	6.2	Q1	U.K.	38	Mitochondrial DNA in inflammation and immunity (33)
2	Jackson MV	2016	Stem Cells	3.6	Q2	U.K.	426	Mitochondrial transfer via tunneling nanotubes is an important mechanism by which mesenchymal stem cells enhance macrophage phagocytosis in the *in vitro* and *in vivo* models of ARDS (39)
3	Picard M	2016	Mitochondrion	4.5	Q1	U.S.	332	The rise of mitochondria in medicine (1)
4	Borcherding N	2023	Nature	48.5	Q1	U.S.	27	The power and potential of mitochondria transfer (2)
5	Saha T	2022	Nature Nanotechnology	35.1	Q1	U.S.	258	Intercellular nanotubes mediate mitochondrial trafficking between cancer and immune cells (7)
6	Xia L	2022	Theranostics	13.3	Q1	China	256	AdMSC-derived exosomes alleviate acute lung injury via transferring mitochondrial component to improve homeostasis of alveolar macrophages (40)
7	Todkar K	2021	Nature Communications	15.7	Q1	Canada	256	Selective packaging of mitochondrial proteins into extracellular vesicles prevents the release of mitochondrial DAMPs (36)
8	Scheiblich H	2021	Cell	42.5	Q1	Germany	254	Microglia jointly degrade fibrillar alpha-synuclein cargo by distribution through tunneling nanotubes (41)
9	Breda CNS	2019	Redox Biology	11.9	Q1	Brazil	222	Mitochondria as central hub of the immune system (42)
10	Puhm F	2019	Circulation Research	16.2	Q1	Austria	221	Mitochondria are a subset of extracellular vesicles released by activated monocytes and induce type I IFN and TNF responses in endothelial cells (43)

IF, Impact Factor (2025); JCR, Journal Citation Reports (2025).

The co-citation timeline further showed a relatively clear evolutionary pattern in the field’s knowledge structure (**Figure 6B**). Early themes were concentrated in clusters such as #2 mitochondrial transfer, #3 tunneling nanotube, #4 central hub, and #7 orchestrating lymphocyte polarity, and were mainly related to intercellular mitochondrial transfer, intercellular connecting structures, and immune cell functional regulation. This was followed by increasing activity in #0 mitochondrial DNA and #1 therapeutic potential, indicating broader attention to mitochondria related signal sensing and disease intervention. In recent years, the most active co-citation clusters included #5 extracellular mitochondria, #6 metabolic rewiring, and #9 harnessing cGAS-STING pathway. Overall, the co-citation network was organized mainly around mitochondria related signal sensing, mechanisms of intercellular transfer, changes in immune cell function, and disease applications.

### 3.7 Mechanistic themes linking mitochondrial transfer to immune regulation

In the keyword network, extracellular vesicles, mesenchymal stem cells, mitochondrial transplantation, T cells, tumor-associated macrophages, and autophagy-associated mitochondrial quality-control terms formed the main topic clusters. Burst terms included mitochondrial transplantation, tunneling nanotube, T cell, mitochondrial biogenesis, mitophagy, and ischemia reperfusion injury. Co-citation analysis identified related knowledge domains involving intercellular mitochondrial transfer, extracellular mitochondria, metabolic rewiring, mitochondrial DNA sensing, and tumor immunity (**Figures 4**–**6**).

Transfer-route terms were represented by tunneling nanotubes, extracellular vesicles, cell-contact-dependent transfer, mitochondrial transplantation, and extracellular mitochondria. These terms corresponded to direct donor-recipient contact, vesicle-associated transfer, uptake of extracellular mitochondria, and exogenous mitochondrial delivery. The studies listed in **Table 2** used different mitochondrial sources, including MSC-derived mitochondria, stromal-cell-derived mitochondria, muscle-derived isolated mitochondria, extracellular vesicle-associated mitochondria, and tumor-associated mitochondrial sources.

**Table 2:** Representative immune-related applications and disease contexts of mitochondrial transfer.

Disease context	Application focus	Mitochondrial source	Transfer route	Recipient system	Immune outcome
Acute graft-versus-host disease with alloimmune inflammation (34)	Treg induction	MSC-derived mitochondria	Direct transfer and artificial mitoception	CD4+ T cells and preclinical inflammatory models	Promotes Treg differentiation and suppressive function, reducing inflammatory responses
Allogeneic Treg cell therapy (44)	Treg functional enhancement	Adipose-derived MSC mitochondria	Coculture-mediated mitochondrial transfer	Human allogeneic Tregs	Improves Treg suppressive activity and supports tolerance-oriented cell therapy
ARDS-associated acute lung injury (39)	Macrophage phagocytic rescue	MSC-derived mitochondria	Tunneling nanotube-mediated transfer	Human and murine macrophage models	Enhances macrophage phagocytosis and supports innate immune clearance
Clinically relevant inflammatory lung injury (45)	Macrophage remodeling	MSC-derived extracellular vesicle mitochondria	Extracellular vesicle-mediated transfer	Human monocyte-derived macrophages and murine alveolar macrophages	Promotes an anti-inflammatory and highly phagocytic macrophage phenotype
LPS-induced acute lung injury (40)	Alveolar macrophage homeostasis	AdMSC exosome-associated mitochondrial components	Exosome-mediated transfer	Alveolar macrophages and LPS-induced lung injury mice	Improves macrophage mitochondrial homeostasis and shifts macrophages toward an anti-inflammatory phenotype
LPS-induced ARDS (46)	Exogenous mitochondrial therapy	Human stem-cell-derived isolated mitochondria	Intravenous mitochondrial transplantation	Alveolar macrophages, pulmonary endothelial cells, and LPS-induced ARDS mice	Reduces inflammatory responses and apoptosis-related lung injury
Asthmatic airway inflammation (47)	T-cell redox regulation	Mitochondria-containing exosomes from airway myeloid-derived regulatory cells	Exosome-mediated mitochondrial transfer	T cells in airway inflammatory models	Transfers mitochondria to T cells and affects bioenergetic and redox responses
Diabetic nephropathy (6)	Macrophage metabolic reprogramming	MSC-derived mitochondria	Coculture-mediated transfer and adoptive transfer	High-glucose macrophages and diabetic nephropathy mice	Promotes macrophage M2-like polarization and restricts renal inflammation
Polymicrobial sepsis (48)	Immune-phase modulation	Isolated mitochondria from L6 muscle cells and umbilical cord MSCs	Intravenous mitochondrial transplantation	Cecal slurry rat model and LPS-stimulated human PBMC monocytes	Improves bacterial clearance and attenuates both hyperinflammation and immune paralysis
Obesity-associated white adipose tissue inflammation (49)	Immunometabolic homeostasis	Adipocyte-derived mitochondria	Endogenous adipocyte-to-macrophage transfer	White adipose tissue macrophages	Defines a mitochondria-acquiring macrophage state and links impaired transfer to obesity-related dysfunction
Cancer immunotherapy (50)	CD8+ T-cell enhancement	Bone marrow stromal cell-derived mitochondria	Ex vivo nanotube-mediated transfer	CD8+ T cells and tumor-bearing mouse models	Improves CD8+ T-cell mitochondrial fitness, tumor infiltration, and antitumor efficacy
Tumor immune evasion (51)	Maladaptive mitochondrial exchange	Tumor-cell-derived mitochondria	Tumor microenvironment-associated mitochondrial transfer	Tumor-infiltrating lymphocytes and cancer models	Contributes to T-cell dysfunction and tumor immune escape

AdMSC, adipose-derived mesenchymal stem cell; ARDS, acute respiratory distress syndrome; BMSC, bone marrow stromal cell; EV, extracellular vesicle; GVHD, graft-versus-host disease; LPS, lipopolysaccharide; MSC, mesenchymal stem cell; PBMC, peripheral blood mononuclear cell; Treg, regulatory T cell.

**Figure 7:**
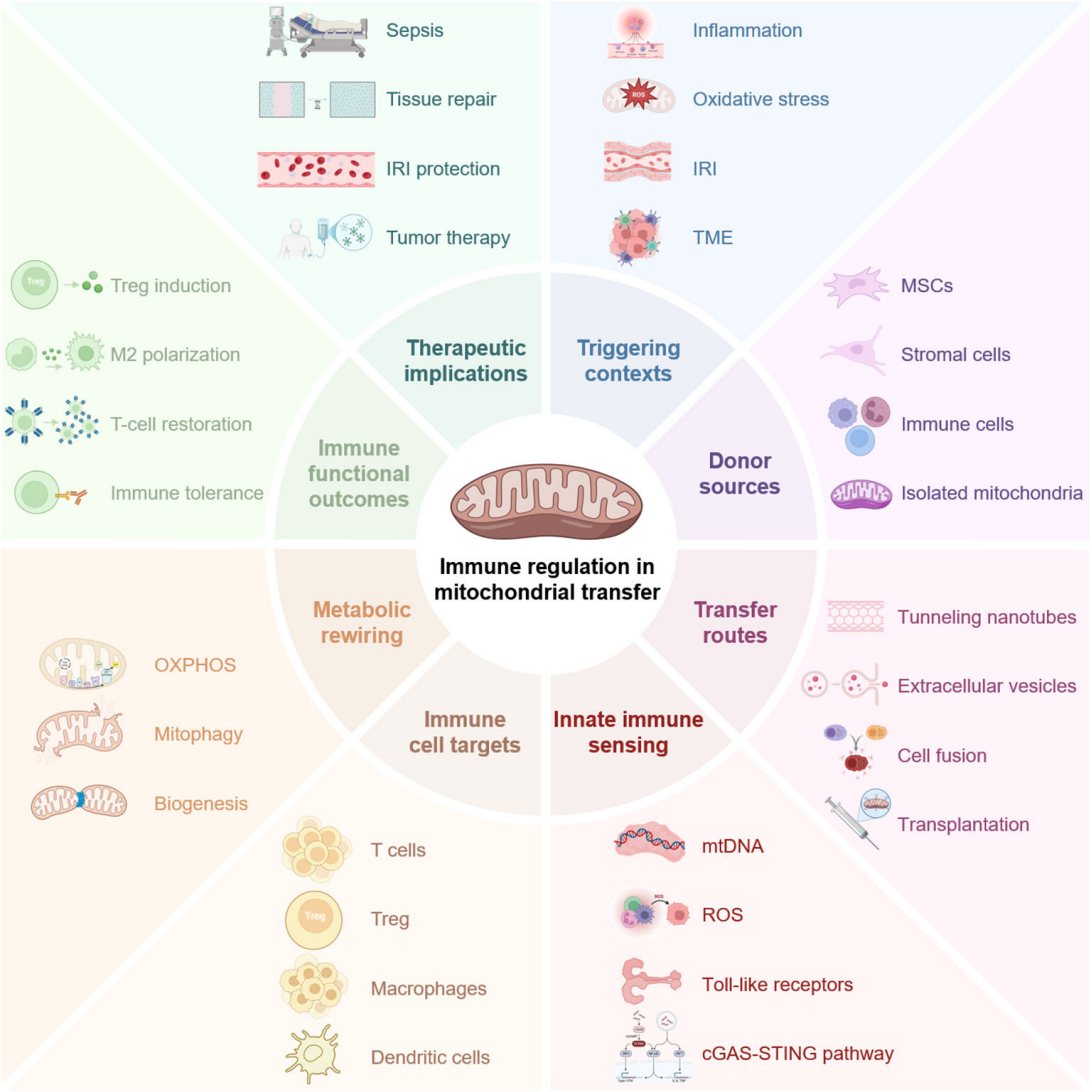
Mechanisms of immune regulation in mitochondrial transfer.

Recipient-cell and disease-related evidence was mainly distributed across macrophage, T-cell, inflammatory-injury, sepsis, and tumor models. Reported macrophage outcomes included enhanced phagocytosis, improved oxidative phosphorylation, reduced inflammatory cytokine production, and M2-like polarization (8, 17). T-cell evidence included Treg differentiation, increased suppressive function, improved CD8^+^ T-cell metabolic fitness, and stronger antitumor activity after mitochondrial transfer. In acute lung injury, ARDS, diabetic nephropathy, and sepsis, the main reported outcomes were bioenergetic recovery, bacterial clearance, reduced inflammatory injury, and organ protection. Tumor-related evidence was less consistent, ranging from enhanced antitumor activity after mitochondrial transfer to CD8^+^ T cells to immune evasion or therapy resistance after mitochondrial exchange in the tumor microenvironment (**Table 2**). **Figure 7** illustrates the main transfer routes and immune-cell responses covered by these representative studies.

## 4 Discussion

### 4.1 Key findings

This bibliometric analysis shows a rapid expansion of research on mitochondrial transfer and immune regulation over the past decade. Publication growth accelerated after 2020, with China and the United States contributing most prominently to the collaboration network. At the topic level, early work was dominated by mtDNA release, damage-associated molecular pattern sensing, and innate immune activation, with mitochondria viewed mainly as sources of inflammatory signals (25, 33). Recent keyword, burst, and co-citation results showed increasing attention to mitochondrial transplantation, tunneling nanotube, extracellular mitochondria, T cells, tumor-associated macrophages, metabolic rewiring, and tumor immunity (34, 50, 52). Representative studies further placed these signals mainly in inflammatory injury, repair-associated recovery, sepsis, and tumor models, with macrophages and T cells as frequent recipient systems (10–12). The main change in the field is a shift from mitochondria as inflammatory signals to mitochondrial transfer as a process linked with recipient-cell metabolism, immune-cell function, and disease microenvironment.

### 4.2 Current insights

The prominence of clusters related to T cells, tumor-associated macrophages, mitochondrial transplantation, and metabolic rewiring suggests that recent work increasingly situates mitochondrial transfer within immunometabolic and microenvironmental contexts (21, 23, 53). As evidence has accumulated across models, attention has focused increasingly on delivery efficiency, intracellular retention, and functional effects after transfer (36, 43, 54). In sepsis and acute inflammatory injury, exogenous mitochondrial delivery has been associated with recovery of ATP supply, the tricarboxylic acid cycle, nucleotide metabolism, and lipid metabolism, indicating effects on immunometabolic regulation (54, 55).

Studies in cancer further show that the outcome of mitochondrial transfer depends on donor origin, recipient cell state, and local context. Transfer from bone marrow stromal cells to CD8^+^ T cells enhance mitochondrial respiration and antitumor activity, whereas dysfunctional mitochondria derived from tumor cells can induce metabolic defects and functional exhaustion in T cells (50, 51).

Across experimental reports, donor source, recipient-cell uptake, intracellular mitochondrial fate, and the duration of functional effects repeatedly influenced the interpretation of immune outcomes (9, 18, 56–58). Donor source may influence both the quality of transferred mitochondria and the immune response that follows. MSC-derived mitochondria are often transferred through direct cell contact, tunneling nanotubes, or extracellular vesicles, and their effects may involve bioenergetic support together with changes in cytokine signaling, macrophage phenotype, phagocytosis, and T-cell suppressive function (34, 39, 44, 45). Isolated mitochondria used for transplantation provide a more direct mitochondrial input, but their activity depends on donor cell or tissue origin, isolation procedure, membrane integrity, mitochondrial DNA integrity, respiratory capacity, and inflammatory potential (56–59). These variables overlap with the main factors affecting cross-study comparison, including donor source, mitochondrial preparation, transfer route, recipient-cell uptake, and post-transfer functional assessment (**Table 3**). Stromal-cell-derived, muscle-derived, and tumor-associated mitochondria may therefore differ in uptake, intracellular handling, and immune consequence (48, 50, 51). After uptake, transferred mitochondria may follow different intracellular paths across models. In human cardiac cells, extracellular mitochondria entered endolysosomal compartments, with many escaping these compartments, associating with the endogenous mitochondrial network, and increasing ATP production (62). In endothelial engraftment models, MSC-derived mitochondria were transferred to endothelial cells through tunneling nanotubes and promoted engraftment through mitophagy (63). In macrophage-related inflammatory models, MSC-to-macrophage mitochondrial transfer activated PGC-1α-mediated mitochondrial biogenesis and PGC-1α/TFEB-mediated lysosome-autophagy, improving macrophage bioenergetics and limiting inflammatory injury (6). The continued prominence of tunneling nanotube, extracellular mitochondria, and mitochondrial transplantation in our analysis is consistent with this shift toward transfer route, mitochondrial fate, and functional readout (36, 39, 43). Standardization and engineering have also received increasing attention. Nomenclature and characterization criteria are beginning to support more consistent comparison of donor source, delivery route, structural integrity, and functional readouts (18, 59). Engineered approaches, including magnetically responsive artificial cells and surface modified mitochondria, support controlled intervention and comparative study (64, 65). Recent interest in T cells, tumor-associated macrophages, immunotherapy, and metabolic reprogramming further suggests increasing emphasis on mechanism and therapeutic relevance (36, 52, 66).

**Table 3:** Methodological challenges in interpreting immune-related mitochondrial transfer studies.

Challenge	Key issue	Reporting need	Representative evidence
Donor-source variability (58, 59)	Mitochondria from different donor cells may differ in viability, immunogenicity, and functional effect	Report donor cell or tissue source, autologous status, allogeneic status, and preparation method	Allogeneic and syngeneic mitochondria can differ in alloreactivity and allorecognition; consensus recommendations also emphasize donor-source reporting
Isolation quality (56, 57)	Rapid isolation may reduce mitochondrial integrity or introduce cellular contaminants	Report isolation method, purity, membrane potential, respiration, and contamination assessment	Differential filtration and optimization of homogenization conditions affect mitochondrial quality for transplantation
Extracellular stability (60)	Isolated mitochondria may lose activity before uptake	Report storage time, carrier system, viability after preparation, and timing of delivery	Hydrogel encapsulation has been used to protect transplanted mitochondria and improve local retention in ischemia-reperfusion models
Targeting and retention (61)	Systemic delivery may have limited accumulation at the intended site	Report delivery route, tissue retention, biodistribution, and off-target uptake	Nanomotorized mitochondria were developed to improve delivery toward ischemic cardiac tissue
Recipient-cell uptake (50)	Detection of mitochondrial signal does not necessarily prove functional integration	Report uptake efficiency, persistence, mitochondrial function, and recipient-cell response	Nanotube-mediated transfer from BMSCs to CD8+ T cells increased mitochondrial respiration and antitumor activity
Maladaptive transfer (51)	Mitochondrial transfer may also support immune dysfunction or disease progression	Report functional outcome after transfer and distinguish protective from harmful effects	Tumor microenvironment-associated mitochondrial transfer can induce T-cell dysfunction and immune evasion
Terminology and comparability (59)	Studies use different terms, assays, and characterization standards	Use consistent nomenclature and minimal characterization criteria	A 2025 Nature Metabolism consensus statement proposed nomenclature and characterization recommendations for mitochondrial transfer and transplantation

DAMPs, damage-associated molecular patterns; EV, extracellular vesicle; MSC, mesenchymal stem cell; OXPHOS, oxidative phosphorylation.

### 4.3 Context-dependent immune outcomes

In sepsis and acute inflammatory injury, mitochondrial interventions have generally been associated with metabolic recovery, reduced inflammatory injury, and improved host defense (45, 46, 55). Metabolomic studies further showed restoration of the tricarboxylic acid cycle, nucleotide metabolism, and lipid metabolism in peripheral blood mononuclear cells and splenocytes in sepsis models, indicating effects on immunometabolic regulation (54). In intracerebral hemorrhage, magnetically responsive artificial cells improved microglial mitochondrial function and promoted immune homeostasis (66). These injury-related models suggest that mitochondrial transfer may be most beneficial when recipient cells show mitochondrial stress, impaired bioenergetics, and excessive inflammatory activation.

In cancer, the effects are less uniform. Enhancing T-cell mitochondrial fitness, modulating tumor-associated macrophage phenotype, and combining mitochondrial intervention with antitumor therapy have each been linked to stronger antitumor immunity (50, 52, 65). In contrast, dysfunctional mitochondria derived from tumor cells can impair T-cell metabolism and function and may promote immune escape (11, 12, 51). The tumor microenvironment adds further complexity because hypoxia, nutrient limitation, suppressive cytokines, metabolic competition, and tumor-derived signals may change how recipient immune cells respond to incoming mitochondria (50, 52, 65). These findings indicate that mitochondrial transfer does not produce a fixed immune effect. Its outcome depends on the combination of mitochondrial source, transfer route, recipient-cell state, and local tissue context.

Future studies should therefore be built around source-defined, recipient-defined, and microenvironment-matched experimental systems. Donor mitochondria need to be characterized by cellular origin, preparation method, membrane integrity, mitochondrial DNA integrity, respiratory capacity, and inflammatory potential (56–59). Recipient immune cells should be stratified by lineage, activation state, mitochondrial damage, metabolic demand, and effector phenotype. Disease models should capture relevant local cues, including cytokine exposure, oxidative stress, hypoxia, nutrient availability, and tumor-associated immune suppression. Experiments comparing the same mitochondrial source across different recipient-cell states, or the same recipient immune cell across inflammatory, repair-associated, and tumor-like microenvironments, may help define when mitochondrial transfer becomes restorative, immunosuppressive, or maladaptive.

### 4.4 What this analysis clarifies

Mitochondrial transfer and immune regulation do not constitute a single line of inquiry. The field includes several related but distinct areas, including mtDNA-associated inflammatory sensing, endogenous mitochondrial transfer, exogenous mitochondrial transplantation, and engineered delivery strategies (25, 33, 34). By placing these topics within the same knowledge map, the present analysis helps define the field’s knowledge base, major shifts in emphasis, and principal research directions, while also clarifying the interpretive scope of different types of evidence.

Our findings further show increasing attention to T cells, the tumor microenvironment, delivery platforms, and standardized characterization, indicating greater emphasis on functional evaluation and potential application (50, 51). To improve cross-study interpretation, future studies should prioritize addressing inconsistent terminology, incomplete characterization of transferred mitochondria after uptake, and poor comparability across donor sources, delivery routes, and disease models (56, 57, 59). In a field expanding this rapidly, conceptual consistency and methodological rigor are as important as the identification of new mechanisms (59).

### 4.5 Limitations

The present analysis reflects the structure of published literature and may be influenced by database coverage, indexing practices, keyword selection, and citation behavior. Although multiple databases were searched and representative experimental studies were considered, some relevant work may have been missed, especially non-English studies or studies using different terminology. Variation in donor source, mitochondrial preparation, transfer route, recipient-cell type, disease model, and outcome measures may also affect cross-study comparison. More consistent reporting and source-specific experimental studies are needed to clarify how mitochondrial transfer shapes immune responses.

## 5 Conclusion

Bibliometric analysis indicates that over the past decade, research on mitochondrial transfer and immune regulation has shifted from mtDNA-associated inflammatory sensing and innate immune activation toward the functional consequences of intercellular mitochondrial transfer, with current emphasis on immunometabolic regulation, T cell function, the tumor immune microenvironment, and delivery strategies. Current evidence further suggests that mitochondrial effects on immune regulation depend on donor source, recipient cell state, and local microenvironment. Further progress will require greater consistency in terminology, characterization, delivery, and cross-model comparison.

## Data Availability

The raw data supporting the conclusions of this article will be made available by the authors, without undue reservation.
