# Supplementary material for: Immune regulation in mitochondrial transfer: knowledge structure and emerging trends from a bibliometric perspective
Source: Front Immunol. 2026 Jun 26;17:1850600. doi: 10.3389/fimmu.2026.1850600 (PMC13349756; doi:10.3389/fimmu.2026.1850600)
Supplement: Supplementary file 1 [file SupplementaryFile1.docx]

***Supplementary Material***

**Table S1** Detailed search strategies and outcomes.

| Database | Query | Results |
| --- | --- | --- |
| **PubMed** | #1: “mitochondrial transfer”[Title/Abstract] OR “intercellular mitochondrial transfer”[Title/Abstract] OR “horizontal mitochondrial transfer”[Title/Abstract] OR “mitochondrial transplantation”[Title/Abstract] OR “mitochondrial delivery”[Title/Abstract] OR “mitochondrial injection”[Title/Abstract] OR “mitochondrial infusion”[Title/Abstract] OR “mitochondrial uptake”[Title/Abstract] OR “mitochondrial exchange”[Title/Abstract] OR “mitochondrial donation”[Title/Abstract] OR “extracellular mitochondria”[Title/Abstract] | 1,957 |
|  | #2: “immunology”[Title/Abstract] OR “immune”[Title/Abstract] OR “immunometabolism”[Title/Abstract] OR “innate immunity”[Title/Abstract] OR “adaptive immunity”[Title/Abstract] | 1,106,341 |
|  | #3: #1 AND #2 | **170** |
| **Embase** | #1: ‘mitochondrial transfer’:ab,ti OR ‘intercellular mitochondrial transfer’:ab,ti OR ‘horizontal mitochondrial transfer’:ab,ti OR ‘mitochondrial transplantation’:ab,ti OR ‘mitochondrial delivery’:ab,ti OR ‘mitochondrial injection’:ab,ti OR ‘mitochondrial infusion’:ab,ti OR ‘mitochondrial uptake’:ab,ti OR ‘mitochondrial exchange’:ab,ti OR ‘mitochondrial donation’:ab,ti OR ‘extracellular mitochondria’:ab,ti | 2,363 |
|  | #2: ‘immunology’:ab,ti OR ‘immune’:ab,ti OR ‘immunometabolism’:ab,ti OR ‘innate immunity’:ab,ti OR ‘adaptive immunity’:ab,ti | 1,494,302 |
|  | #3: #1 AND #2 | **205** |
| **Cochrane** | #1: (mitochondrial transfer):ab,ti,kw OR (intercellular mitochondrial transfer):ab,ti,kw OR (horizontal mitochondrial transfer):ab,ti,kw OR (mitochondrial transplantation):ab,ti,kw OR (mitochondrial delivery):ab,ti,kw OR (mitochondrial injection):ab,ti,kw OR (mitochondrial infusion):ab,ti,kw OR (mitochondrial uptake):ab,ti,kw OR (mitochondrial exchange):ab,ti,kw OR (mitochondrial donation):ab,ti,kw OR (extracellular mitochondria):ab,ti,kw | 819 |
|  | #2: (immunology):ab,ti,kw OR (immune):ab,ti,kw OR (immunometabolism):ab,ti,kw OR (innate immunity):ab,ti,kw OR (adaptive immunity):ab,ti,kw | 74,927 |
|  | #3: #1 AND #2 | **43** |
| **Scopus** | #1: TITLE-ABS-KEY (“mitochondrial transfer” OR “intercellular mitochondrial transfer” OR “horizontal mitochondrial transfer” OR “mitochondrial transplantation” OR “mitochondrial delivery” OR “mitochondrial injection” OR “mitochondrial infusion” OR “mitochondrial uptake” OR “mitochondrial exchange” OR “mitochondrial donation” OR “extracellular mitochondria”) | 2,280 |
|  | #2: TITLE-ABS-KEY (“immunology” OR “immune” OR “immunometabolism” OR “innate immunity” OR “adaptive immunity”) | 2,262,147 |
|  | #3: #1 AND #2 | **224** |
| **Web of science** | #1: TS = (mitochondrial transfer OR intercellular mitochondrial transfer OR horizontal mitochondrial transfer OR mitochondrial transplantation OR mitochondrial delivery OR mitochondrial injection OR mitochondrial infusion OR mitochondrial uptake OR mitochondrial exchange OR mitochondrial donation OR extracellular mitochondria) | 71,137 |
|  | #2: TS = (immunology OR immune OR immunometabolism OR innate immunity OR adaptive immunity) | 1,230,466 |
|  | #3: #1 AND #2 | **3,038** |

**Table S2** Top ten institutions publishing research on mitochondrial transfer and immunity.

| **Rank** | **Institutions** | **Publication count** | **Citation count** | **Countries** |
| --- | --- | --- | --- | --- |
| 1 | Sichuan University | 87 | 75 | China |
| 2 | Harvard Medical School | 48 | 215 | United States |
| 3 | University of Pittsburgh | 48 | 17 | United States |
| 4 | Shanghai Jiao Tong University | 39 | 5 | China |
| 5 | Zhejiang University | 33 | 14 | China |
| 6 | Sun Yat-sen University | 31 | 39 | China |
| 7 | Central South University | 30 | 45 | China |
| 8 | China Medical University | 30 | 40 | China |
| 9 | Université Laval | 28 | 185 | Canada |
| 10 | Fudan University | 27 | 28 | China |

**Table S****3** Top ten authors by citation count in mitochondrial transfer and immunity research.

| **Rank** | **Authors** | **Citation count** | **Publication count** |
| --- | --- | --- | --- |
| 1 | Caicedo, A | 71 | 9 |
| 2 | Khoury, M | 58 | 8 |
| 3 | Luz-Crawford, P | 57 | 3 |
| 4 | Boilard, E | 56 | 6 |
| 5 | O'Kane, CM | 53 | 1 |
| 6 | Kissenpfennig, A | 53 | 1 |
| 7 | Matthay, MA | 53 | 1 |
| 8 | Mcauley, DF | 53 | 1 |
| 9 | Doherty, DF | 53 | 1 |
| 10 | Morrison, TJ | 53 | 1 |

**Table S4** Top ten funding agencies supporting mitochondrial transfer and immunity research.

| **Rank** | **Funding Agencies** | **Publication count** | **Countries** | **Proportion** |
| --- | --- | --- | --- | --- |
| 1 | National Natural Science Foundation of China | 255 | China | 26.37% |
| 2 | United States Department of Health and Human Services | 174 | United States | 17.99% |
| 3 | National Institutes of Health | 173 | United States | 17.89% |
| 4 | Instituto Politécnico Nacional | 72 | Mexico | 7.45% |
| 5 | German Research Foundation | 46 | Germany | 4.76% |
| 6 | National Heart, Lung, and Blood Institute | 38 | United States | 3.93% |
| 7 | National Institute of Allergy and Infectious Diseases | 34 | United States | 3.52% |
| 8 | National Cancer Institute | 33 | United States | 3.41% |
| 9 | National Key Research and Development Program of China | 25 | China | 2.59% |
| 10 | Ministry of Education, Culture, Sports, Science and Technology, Japan | 24 | Japan | 2.48% |

**Table S5** Top ten journals publishing research on mitochondrial transfer and immunity.

| **Rank** | **Journal** | **IF** | **JCR** | **Publication count** | **Citation count** |
| --- | --- | --- | --- | --- | --- |
| 1 | Frontiers in Immunology | 5.9 | Q1 | 75 | 59 |
| 2 | International Journal of Molecular Sciences | 4.9 | Q1 | 32 | 35 |
| 3 | Advanced Science | 14.1 | Q1 | 16 | 23 |
| 4 | Biomaterials | 12.9 | Q1 | 14 | 7 |
| 5 | Cells | 5.2 | Q2 | 13 | 25 |
| 6 | Nature Communications | 15.7 | Q1 | 12 | 42 |
| 7 | Scientific Reports | 3.9 | Q1 | 12 | 11 |
| 8 | Frontiers In Cell and Developmental Biology | 4.3 | Q1 | 11 | 16 |
| 9 | Advanced Functional Materials | 19.0 | Q1 | 10 | 0 |
| 10 | Cell Reports | 6.9 | Q1 | 10 | 6 |

IF: Impact Factor (2025); JCR: Journal Citation Reports (2025).
